# Crosslinked Polyimide and Reduced Graphene Oxide Composites as Long Cycle Life Positive Electrode for Lithium‐Ion Cells

**DOI:** 10.1002/cssc.202001389

**Published:** 2020-09-02

**Authors:** Hui Gao, Bingbing Tian, Haofan Yang, Alex R. Neale, Marc A. Little, Reiner Sebastian Sprick, Laurence J. Hardwick, Andrew I. Cooper

**Affiliations:** ^1^ Materials Innovation Factory and Department of Chemistry University of Liverpool 51 Oxford St Liverpool L7 3NY UK; ^2^ International Collaborative Laboratory of 2D Materials for Optoelectronics Science and Technology of Ministry of Education Institute of Microscale Optoelectronics Shenzhen University Shenzhen 518060 P. R. China; ^3^ Stephenson Institute for Renewable Energy Department of Chemistry University of Liverpool Peach St Liverpool L69 7ZD UK

**Keywords:** Cathode, composites, crosslinked polyimides, lithium-ion batteries, long cycle life

## Abstract

Conjugated polymers with electrochemically active redox groups are a promising class of positive electrode material for lithium‐ion batteries. However, most polymers, such as polyimides, possess low intrinsic conductivity, which results in low utilization of redox‐active sites during charge cycling and, consequently, poor electrochemical performance. Here, it was shown that this limitation can be overcome by synthesizing polyimide composites (PIX) with reduced graphene oxide (rGO) using an in situ polycondensation reaction. The polyimide composites showed increased charge‐transfer performance and much larger specific capacities, with PI50, which contains 50 wt % of rGO, showing the largest specific capacity of 172 mAh g^−1^ at 500 mA g^−1^. This corresponds to a high utilization of the redox active sites in the active polyimide (86 %), and this composite retained 80 % of its initial capacity (125 mAh g^−1^) after 9000 cycles at 2000 mA g^−1^.

## Graphical abstract

**Live long and prosper**: Composites of reduced graphene oxide and a three‐dimensional polyimide with electrochemical redox active groups are synthesized and used as positive electrodes in lithium‐ion batteries. The composites show higher specific capacity (125 mAh g^−1^, 2000 mA g^−1^) and longer cycling life (80 % retention after 9000 cycles) than the polyimide on its own.

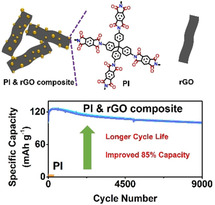

## Introduction

Lithium‐ion batteries are important in portable electronic devices and electric vehicles.^1^, ^2^, ^3^ Organic materials have attracted attention as candidate lightweight electrodes for next generation lithium‐ion batteries.^4^, ^5^ They can deliver high capacity because of a high density of active sites per mass unit and can in principle be obtained from biomass‐ derived resources using low temperature synthesis routes. The structure of organic molecules can also be tailored to give specific electrochemical properties. To date, conductive polymers,^6^, ^7^ organosulfur compounds,^8^, ^9^, ^10^ nitroxyl radical‐ bearing compounds,^11^, ^12^ conjugated carbonyls‐containing compounds,^13^, ^14^, ^15^, ^16^ imine‐functionalized compounds,^17^, ^18^ and azo‐functionalized molecules^19^, ^20^ have been used as positive electrode materials in lithium‐ion batteries. Conjugated carbonyl compounds are particularly promising candidates because they undergo reversible redox reactions with lithium ions to form an intermediate with a delocalized change at potential around 2.5 V vs. Li^+^/Li.^21^ For example, pyrene‐4,5,9,10‐tetraone undergoes a four‐electron reduction process at an average potential of 2.59 V vs. Li^+^/Li and reaches a high reversible capacity of 360 mAh g^−1^_._ 13 This capacity is more than twice the value of LiCoO_2_ (albeit at a discharge potential that is more than 1 V lower than for LiCoO_2_).^13^ Consequently, molecules containing a high content of conjugated carbonyl groups are a focus of current research in organic electrode materials. However, there are challenges associated with these types of organic electrode material, including the inefficient utilization of the redox‐active carbonyl sites, particularly at high current rates and during long‐term operation, which restricts their application.

The utilization of active sites in electrochemically redox‐active organic materials is affected by the dissolution of active material in the electrolyte, the chemical stability of active material during cycling, and the ion and electron transport capability of the organic electrode. The dissolution of the small organic active material in the electrolyte can be suppressed by various strategies, including grafting the electroactive molecule into a conductive backbone,^22^ forming a salt,^23^ and synthesizing insoluble polymeric materials.^24^ Polymeric materials are advantageous because their molecular structures can be chemically tuned based on the employment of different monomers to improve their performance.^25^ Polymerization is also a good strategy to prevent the dissolution of active material during cycling. Recent reports have shown that polymers containing conjugated carbonyl groups show better capacity retention over hundreds of cycles than the corresponding monomers.^26^, ^27^

Conductive coatings, and the incorporation of conductive additives, have been used to enhance charge transport in organic electroactive materials, and these strategies have been shown to improve rate capability.^28^, ^29^, ^30^ For example, a microporous polyimide covalent‐organic framework and carbon nanotube (CNT) composite that was prepared by in situ polymerization was reported to exhibit a near 100 % capacity retention after 8000 cycles, 95 mAh g^−1^ at 2000 mA g^−1^ (19.2 C), and 104 mAh g^−1^ at 200 mA g^−1^ (1.9 C).^31^ Taken together, these results suggest that covalent‐organic framework composites can be made to be highly stable and to undergo fast redox reactions.

**Scheme 1:**
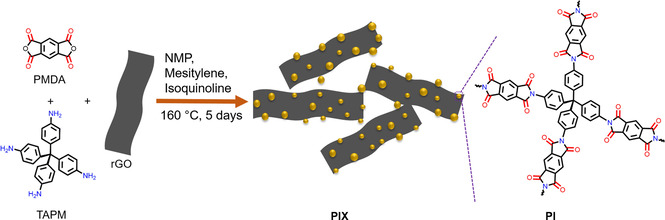
**S**ynthesis of the PMDA‐based polyimide polymer network.

Here, we study composites of a 3D redox‐active polyimide (PI, Scheme 1) and reduced graphene oxide (rGO) that were synthesized using in situ polymerization of pyromellitic dianhydride (PMDA) and tetra‐(4‐aminophenyl)methane (TAPM). By itself, PI was found to have a low specific capacity of 3 mAh g^−1^ as the active material in a lithium‐ion half‐cell, indicating low utilization of the redox‐active groups in PI. However, upon the addition of rGO during in situ polymerization of PI, a significant improvement in performance is observed, with the PI composite containing 50 wt % of rGO having a 86 % utilization of the redox‐active sites and a specific capacity of 172 mAh g^−1^ at 500 mA g^−1^. The improvement in measured specific capacity is explained by changes to the surface area and charge‐transport of the composites relative to the polyimide material by itself.

## Results and Discussion

A redox‐active polyimide (PI) was synthesized according to a reported polycondensation procedure.^32^ PI was synthesized by dispersing PMDA and TAPM in a stoichiometric molar ratio in a mixture of *N*‐methyl‐2‐pyrrolidone (NMP), mesitylene, and isoquinoline, followed by heating the reagents at 160 °C for 5 days to afford the product in high yield (typically >90 %; Scheme 1). Here, PI and the PI composites were synthesized using the same method, but 10, 30, and 50 wt % rGO was added into the reaction mixture with PMDA and TAPM during the synthesis (see Experimental Section for detailed synthesis,). The as‐prepared PI and PI composites (PI*X*, where *X*=10, 30, and 50 wt %) are insoluble in water and common organic solvents, such as acetone, ethanol, *n*‐hexane, tetrahydrofuran, and *N*,*N’*‐dimethylformamide.

**Figure 1:**
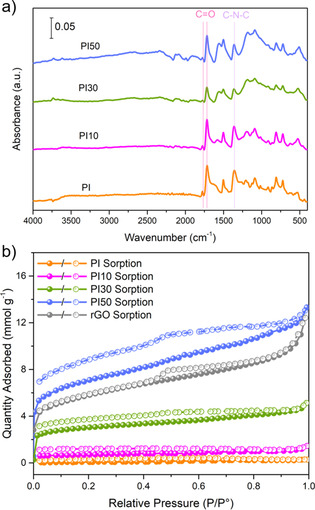
a) FTIR spectra of PI and the PI*X* composites. b) N_2_ sorption isotherms of PI, the PI*X* composites, and rGO (77.3 K); filled circles, adsorption experiments; unfilled circle, desorption experiments.

**Figure 2:**
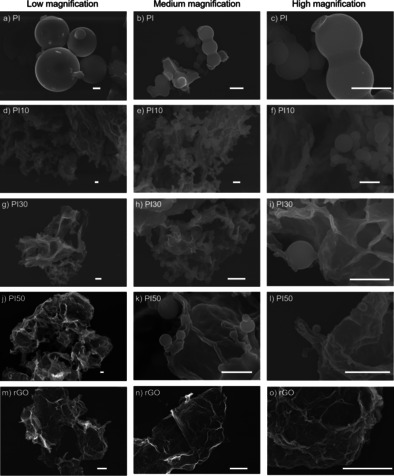
SEM images of (a–c) PI; (d–f) PI10; (g–i) PI30; (j–l) PI50, and (m–o) rGO (scale bar: 1 μm).

The as‐prepared PI and PI*X* were characterized by Fourier‐transform infrared spectroscopy (FTIR) (Figure 1a). The FTIR spectra of the PMDA, TAPM, and rGO precursors are shown in Figure S1 in the Supporting Information. The IR stretching vibrations of the −NH_2_ groups in TAPM, at around 3300 cm^−1^, are not present in the IR spectra of the composite samples, indicating that TAPM has undergone polycondensation with PMDA. Bands at 1772 and 1719 cm^−1^, attributed to asymmetric and symmetric stretching vibrations of the C=O groups of the five‐membered imide rings, were observed in the IR spectra of the PI*X* composite samples, as was the band at 1356 cm^−1^, assigned to C−N−C moiety. The bands at 1566 and 1191 cm^−1^ in the spectra of the PIX composites had increased in intensity as a function of rGO content and were assigned to the stretching vibrations of the C=C and C−O−C bonds in rGO. Scanning electron microscopy (SEM) was used to study the morphology of PI and the PI*X* composites, which showed predominantly spherical particles for pristine PI, and spherical particles on the edge of the rGO in the PI*X* composites (Figure 2). However, the size of the polyimide spheres differs in the composites and the particle size decreases with the increase in rGO content in the composite (Figure 2). PI on its own shows a diameter range of 1.0 to 7.0 μm. By contrast, PI10, PI30, and PI50 have diameter ranges of 0.2–1.4, 0.2–1.2, and 0.1–0.8 μm, respectively. Powder X‐ray diffraction (PXRD) patterns show that PI, rGO, and the PI*X* composites are amorphous (Figure S2).

N_2_ sorption isotherms at 77.3 K were used to determine the adsorption behavior of PI and the PI*X* composites. The N_2_ isotherms for PI and PI10 show that PI is effectively non‐porous (Figure 1b) and has a N_2_ sorption capacity of 0.29, and that PI10 has a low N_2_ sorption capacity of 1.45 mmol g^−1^ at *P*/*P* ^0^=1.0 (Figure 1b, Table S1). By contrast, the N_2_ isotherms of PI30 and PI50 show that these samples are porous and have gas uptakes in the micro‐, meso‐, and macroporous regimes. Pore size distributions for PI30 and PI50 were determined by non‐local density functional theory (NL‐DFT; Figure S3) and indicate the presence of micropores, and to a lesser extent, meso‐ and macropores. Brunauer‐Emmett‐Teller surface areas (*SA*_BET_) for the composites increased as the wt % of rGO content was increased from 52 m^2^ g^−1^ for PI10 up to 563 m^2^ g^−1^ for PI50 (Table S1), which can be partly explained by the unreacted rGO that was used in this study having a *SA*_BET_ of 439 m^2^ g^−1^. The observation that PI is non‐porous to N_2_ indicates that the polymer networks are interpenetrated, which is often observed for crosslinked porous polymers.^33^, ^34^ Therefore, it appears that the introduction of rGO in the PI*X* composites, increases the surface area of the composite, which has previously been observed by others.^35^ Both PI30 and PI50 have pores with an average width of 15 Å as determined by NL‐DFT, which is larger than the diameter of the lithium ion (1.52 Å) and could, therefore, be beneficial for lithium‐ion transport within the material.

Next, electrodes were fabricated to evaluate the electrochemical performance of PI and the PI*X*. Coin cells were prepared using PI/PI*X* as the working positive electrode, lithium as the counter electrode and 1 m lithium bis(trifluoromethanesulfonyl)imide (LiTFSI) in 1,3‐dioxolane (DOL) and 1,2‐dimethoxyethane (DME) (1 : 1 *v*/*v*) as the electrolyte. This electrolyte formulation was selected because it has been reported that the capacity of organic electrodes fades more rapidly in conventional Li‐ion battery electrolytes (based on lithium hexafluorophosphate in organic carbonates) than those tested in LiTFSI‐DME/DOL‐based electrolytes.^26^, ^36^

**Figure 3:**
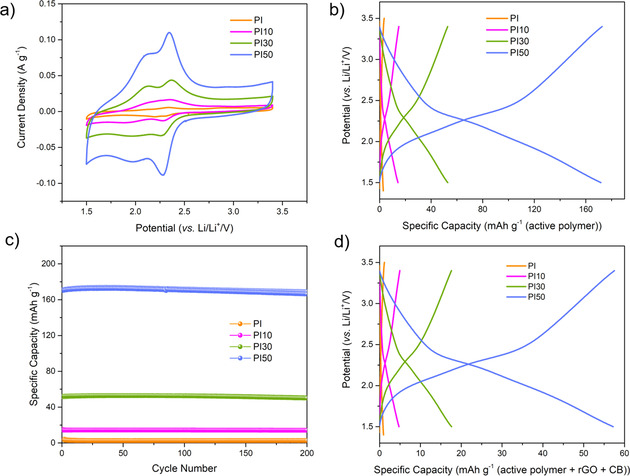
a) CV profiles at a scan rate of 0.1 mV s^−1^. b) Charge‐discharge profiles at 500 mA g^−1^. c) Cycling performances over 200 cycles at 500 mA g^−1^. d) Charge‐discharge profiles [based on active polymer, rGO, and carbon black (CB)] at 500 mA g^−1^ for PI, PI10, PI30, and PI50.

Cyclic voltammetry (CV) was performed using the PI and PI*X* coin cells at 0.1 mV s^−1^ (Figure 3a), with all the samples exhibiting similar shaped CV characteristics. Two pairs of redox peaks corresponding to the two‐electron reaction of the carbonyl groups in one PMDA unit were centered at approximately 2.3 and 2.0 V.^24^ Furthermore, the peak current densities and integral charge (as normalized to the mass unit of PI active material) increased as the mass ratio of rGO in the PI*X* composite was increased. PI has the smallest integral charge area, when compared with the PI*X* composites, showing low utilization of redox‐active sites in PI on its own. The smaller particle sizes of PI in the PI*X* composites mean that they have a larger surface area/volume ratio, which might enhance the number of exposed redox‐sites in PI. Consequently, the size difference induced by introduction of rGO appears to improve Li^+^ ion diffusion to the redox‐active carbonyl groups of PI and, thus, increases their utilization. It is also possible that rGO in the composites enhances charge‐transport within these materials, which is also beneficial.

Galvanostatic charge/discharge experiments were performed in a voltage window of 1.5 to 3.4 V for all positive electrodes (Figure 3b,d). At a current density of 500 mA g^−1^, PI has a limited capacity of 3 mAh g^−1^, which is only 2.2 % of the maximum theoretical specific capacity for PI (144 mAh g^−1^, see Figure S5 for calculation details). This low utilization of its electrochemical redox‐active sites is likely due to the poor electrical conductivity of PI, and non‐accessible redox sites due to the inherent non‐porosity of the polymer. Indeed, it was found that the composites (containing rGO) show higher capacities of 14, 53, and 172 mAh g^−1^ at 500 mA g^−1^ for PI10, PI30, and PI50, respectively. While rGO can contribute to the capacity of the PI*X* composites (see below), rGO can only contribute up to 5, 21, and 48 mAh g^−1^ to the specific capacities of PI10, PI30, and PI50, respectively (Equation S2 and Table S2). Hence, the utilization of redox‐active sites, compared to the theoretical specific capacity, increases significantly to 6.2, 22, and 86 % for PI10, PI30, and PI50, respectively. Moreover, PI*X* materials exhibited good cycle life performance and capacity retentions after 200 cycles are 93, 96, and 98 % for PI10, PI30, and PI50 (Figure 3c).

The capacity of PI50 is comparable to typical positive electrode materials used in commercial cells, such as LiCoO_2_ which has a capacity of 145 mAh g^−1^ and a cycle life of 1000, when considering the active material.^37^ However, when the capacity of the PI50 cell is calculated based on summing mass of the active material and conductive additives it was found to be only 52 mAh g^−1^ (Figure 3d). Here, all gravimetric capacities and currents are normalized to the mass of the active material (PI), with the exception Figure 2d, wherein the galvanostatic charge/discharge profiles are based on the mass of the whole electrode in the cell.

Next, we explored whether the intricate mixture of the PI*X* composites was required to obtain the observed increase in performance. For this, a physical mixture of PI with rGO (PI/rGO) was prepared by ball milling a 1 : 1 mass ratio of PI and rGO. When this mixture was used as active component the resulting cell had a capacity of 86 mAh g^−1^ (Figure S6) at 500 mA g^−1^, which is 70 % lower than PI50, which was synthesized by in situ polycondensation. The physical mixture also has lower capacity retention of 78 % after 200 cycles (Figure S7). The additives used in the cells – that is, rGO and carbon black – were also tested for their electrochemical performance, to evaluate their respective contributions towards the overall performance of the cells. When tested under the same conditions rGO/PVDF (9 : 1 by mass) and carbon black: PVDF (9 : 1 by mass) had a specific capacity of 48 and 1 mAh g^−1^ (Figures S8–S11) originating primarily from capacitive charging of the electrochemical double layer.

**Figure 4:**
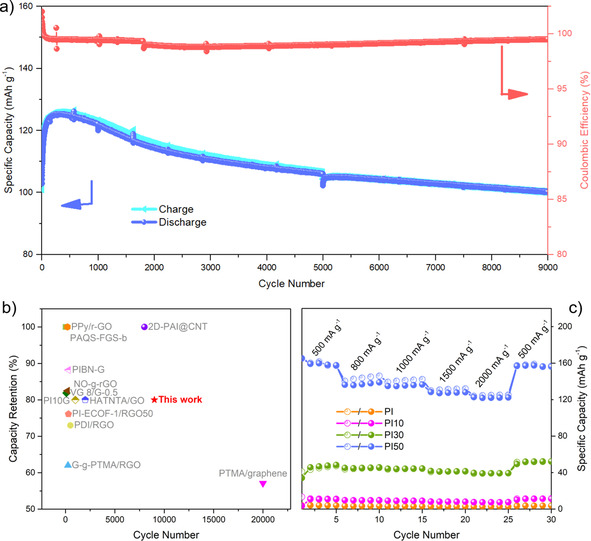
a) Long‐term cycling stability of PI50 at 2000 mA g^−1^. b) Comparison of the cycling performance to other polyimide cathodes (reference information for comparative data shown in the SI). c) Rate performance of PI, PI10, PI30, and PI50. Please note that the break in the coulombic efficiency plot is due to a break in the cycling measurement; unfilled circles, charge experiments; filled circles, discharge experiments.

Cycle life is another important factor for the performance of a battery cell, in addition to the specific capacity of the material. The long‐term cycling performance of PI50 was studied at 2000 mA g^−1^ (Figure 4a). A capacity of over 125 mAh g^−1^ was observed for PI50 during this measurement, and minor capacity loss was observed for a PI50 during 9000 charge‐discharge cycles (capacity loss of *ca*. 0.0027 % per cycle, 80 % retention of the highest recorded capacity), demonstrating stable active site utilization and excellent rate performance. The coulombic efficiency for PI50 was also maintained at close to 100 %, at a current density of 2000 mA g^−1^ (≈13.8 C). The cycling performance of PI50 outperforms a series of organic hybrids materials, prepared using graphene, rGO, and CNTs (Figure 4b and Table S3). For example, PI‐ECOF‐1/rGO50 (where ECOF=exfoliated covalent‐organic framework) was reported to have a 76 % capacity retention after 300 charge‐discharge cycles under 1 C,^26^ PIBN‐G (where PIBN‐G=a microporous two‐dimensional poly(imide‐benzoquinone) covalent‐organic framework composite with graphene) exhibited 88 % of the initial capacity after 300 charge‐discharge cycles under 5 C,^14^ and 2D‐PAI@CNT (where PAI=polyarylimide) showing the highest cycling performance reported (at 100 % retention after 8000 charge‐discharge cycles at 20 C).^31^ In this context, it is important to note that long‐term testing is required to make fair conclusions regarding materials performances, which is unfortunately often not reported.

To gain insight into the potential structural/chemical changes of PI50 after the long‐term cycling test, FTIR spectra were measured for the as‐made material and for electrode material that was recovered from a cell that underwent 500 charge‐discharge cycles at 500 mA g^−1^. For the cycled material, the coin cell was disassembled inside a glovebox and the electrode was washed with DOL before being dried under reduced pressure at room temperature. The FTIR measurements were then performed under a nitrogen atmosphere. The FTIR spectra of the PI50 electrodes before and after 500 cycles showed that the characteristic carbonyl (1772 and 1718 cm^−1^) and C−N−C (1356 cm^−1^) bands were retained after cycling (Figure S12). SEM images taken after the cycling experiment showed no obvious changes to the morphology of PI50 after 500 cycles (Figure S13).

The rate performance of PI and the PI*X* composites were investigated at different current densities (Figure 4c) and found to increase over the current rate range from 500–2000 mA g^−1^ (3.5 to 13.9 C). Reversible capacities for PI50 of 165, 145, 142, 132, and 125 mAh g^−1^ were determined at 500, 800, 1000 1500, and 2000 mA g^−1^, respectively. In addition, the capacity of 165 mAh g^−1^ for PI50 recovered to 159 mAh g^−1^ after 20 cycles at different currents, corresponding to 96 % of the initial capacity at 500 mAh g^−1^, implying good rate performance. PI and the PI*X* composites were calculated to have good coulombic efficiencies (around 100 %) at the different scan rates (Table S4). Initial fluctuations and coulombic efficiencies marginally greater than 100 % indicated additional reactions occurred during the early cycles. However, these stabilized with continued cycling and did not appear to negatively affect cell stability; for example, PI50 could be cycled 9000 times (Figure 4a). The rate performance of PI50 is better than many other reported polyimide electrode materials; for example, PI‐ECOF‐1/rGO30 and PI‐ECOF‐1/rGO50 discharged at 10 C still retain capacities of 60 and 90 mAh g^−1^,^26^ while DAAQ‐ECOF (where DAAQ=2,6‐diamino‐anthraquinone) has a capacity of 87 mAh g^−1^ at a current density of 2000 mA g^−1^, which is equivalent to 13.2 C.^38^

**Figure 5:**
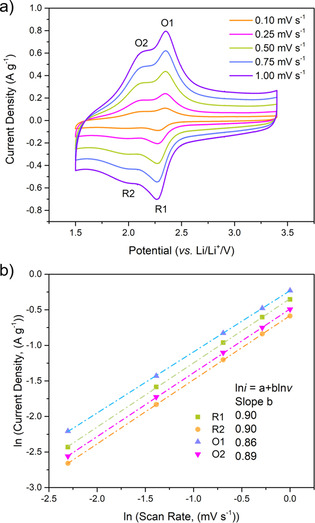
a) CVs of PI50 collected at different scan rates. b) ln *i* vs. ln V plots to determine the b‐values of different peaks.

To further understand the rate capability, the electrochemical reaction kinetics of PI50 were investigated by scanning the CV at different sweep rates (Figure 5). The current response (*i*) of PI50 to the applied sweep rate (*v*, 0.1–1.0 mV s^−1^) was measured (Figure 5a). According to the power law, *i*=a*v* ^b^, b‐values close to 0.5 indicate that the current is controlled by semi‐infinite linear diffusion, by contrast, b‐values close to 1 indicate that the current is surface‐controlled. The b‐values of four redox peaks R1, R2, O1, and O2 (0.90, 0.90, 0.86, and 0.89) are all close to 1 (Figure 5b), indicating that the charge storage in PI50 is a fast surface‐controlled process, which is expected as storage is through reversible redox‐active functional groups. To account for some of the dominant capacitive contributions from the rGO, the equivalent peak analysis was performed following baseline subtractions from linear back extrapolation of the positive and negative currents baselines in the region of approximately 2.8–3.2 V vs. Li^+^/Li. Consequently, the derived b‐values for R1, R2, O1, and O2 reduced only slightly (0.8, 0.74, 0.84, 0.85, respectively, Figure S14), further supporting the nature of the surface‐controlled processes on the active polymer. Further analysis of the voltammetric sweep rate dependence enables the surface controlled contribution to the current response to be quantitatively distinguished.^31^, ^39^ We can express the current response at a fixed potential as being the combination of two separate mechanisms, surface‐controlled contribution and diffusion‐controlled insertion processes. *i*=*k*_1_ *v*+*k*_2_ *v* ^1/2^, the exact surface‐controlled contribution (k_1_ *v*) can be further quantified, where *k*_1_ and *k*_2_ constants can be simulated by plotting *iv* ^−1/2^ versus *v* ^1/2^, in which *k*_1_ and *k*_2_ are the slope and intercept of the linear fitting plot. At 0.1 mV s^−1^, the surface contribution is determined to be 68 %, which is further enhanced to 87 % at 1 mV s^−1^.

Electrochemical impedance spectroscopy (EIS) was employed to further characterize the electrochemical and transport behavior of PI and the PI*X* composites (Figure S15a). A charge‐transfer resistance value (*R*_c_) was obtained by fitting Nyquist plots using an equivalent circuit model (Figure S15b). In this circuit, the constant phase element (CPE), *R*_c_, the resistance of the solution (*R*_s_), and the mass‐transport resistance (*W*) were considered. The CPE is in parallel to the resistance *R*_c_ and *W*, and they are connected in series to *R*_s_. The semicircles in high‐frequency regions denote *R*_c_, which is related to reaction kinetics, and the linear increase in the low‐frequency regions represents Li^+^‐diffusion resistance in the electrolyte. Fitted values for the individual elements in the equivalent circuit of the EIS data of PI and the PI*X* composite electrodes are summarized in Table S5. It was found that that the *R*_c_ of PI50 (22.1 Ω) is smaller than that of PI (47.4 Ω), PI10 (38.1 Ω), and PI30 (35.4 Ω), supporting the assertion of improved charge transfer in PI50 due to optimization of contact between the PI and conductive carbon network.

**Figure 6:**
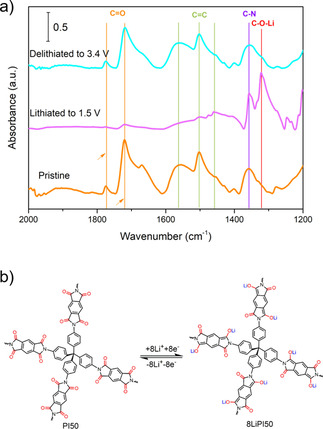
a) FTIR Spectra of PI50 electrode materials at different states of lithiation/delithiation (pristine, lithiated to 1.5 V and delithiated to 3.4 V). b) Proposed reversible electrochemical redox mechanism of PI50 during the lithiation/delithiation process.

The mechanism of the lithiation and delithiation of conjugated carbonyl containing compounds has not been well‐studied. It is important to gain insight into the mechanism to aid further material discovery and for this, we used ex situ FTIR spectroscopy to understand the redox processes. The underlying redox processes of the polyimide in the presence of lithium ions were investigated using ex situ FTIR spectroscopy (Figure 6a). For this, cells with PI50 as the active material were disassembled at the end of the lithiation and delithiation stages, washed by DOL and dried under reduced pressure at room temperature, and FTIR spectra were then recorded. It was found that for the pristine electrode materials, the IR band at 1497 and 1579 cm^−1^ can be assigned to the vibration of C=C bonds, and peaks at 1718 and 1772 cm^−1^ assigned to the vibration of the C=O bonds in PI50. When the sample was discharged to 1.5 V (lithiation), a new band at 1450 cm^−1^ became visible and this band is attributed to the vibration of C=C bonds. A new band at 1313 cm^−1^ can be assigned to the vibration of C−O bonds, indicating the formation of new C=C bonds and C−O−Li bonds in lithiated PI50. The signal at 1718 and 1772 cm^−1^ relating to C=O bonds is weaker after discharge to 1.5 V. When charged back to 3.4 V, the band at 1450 and 1313 cm^−1^ disappeared and the signal at 1718 and 1772 cm^−1^ returned to a state similar to that of the pristine sample, implying that the lithiation/delithiation process is reversible under these conditions. The fact that the IR signal intensity for carbonyl groups on the polyimide is reduced, but still a low‐intensity band remains visible indicates that not full, but rather a partial lithiation of the polyimide C=O bonds occurs during the discharging process, in line with the observed electrochemical performance. The reversible changes of the C=O and C=C bonds vibrational modes in the electrode agree with the lithiation/delithiation of carbonyls in PI50, which is evidence for participation of C=O double bonds in the redox reaction.

Based on these results we suggest a mechanism as outlined in Figure 6b that involves redox reactions of carbonyl units in PI. This is in line with previous reports that have also suggested that only two carbonyl groups of dianhydride PMDA moieties participate in the redox reactions due to the electrostatic repulsion between injected negative charges.^15^, ^40^

## Conclusions

PI*X* (polyimide, where *X*=10, 30, and 50 wt %) composite materials were synthesized by in situ polycondensation on reduced graphene oxide (rGO) and used as redox‐active positive electrode material in a lithium‐ion cell. The reduced particle size of the polyimide polymer in the composites from this synthesis route resulted in the dramatic enhancement of the accessible electrochemically active surface area and, in turn, improved both electron transfer and Li^+^ ion transport to the redox‐active carbonyl groups. As a consequence, the electrochemical performance of PI was improved and the composite materials were found to have much greater specific capacities, with PI50 having the largest of 172 mAh g^−1^ at 500 mA g^−1^. This corresponded to high utilization of the redox active sites of 86 %, and a retention of 80 % of the capacity of 125 mAh g^−1^ after 9000 cycles at 2000 mA g^−1^. Detailed Fourier‐transform (FT)IR investigations showed conclusively the mechanism of storage on the PI resulted from the participation of C=O double bonds in the redox reaction.

The use of redox active functional groups in polymers as a high‐rate, long‐life energy‐storage mechanism shows promise. However, the relative amount of redox‐active material needs to be considered, which is thus far too low for practical applications. This is because of the significant ratio of conductive additives that are used. Looking forward, we need to optimize these materials in terms of their accessible redox‐active sites while lowering the carbon loadings.

## Experimental Section

**Materials**: Pyromellitic dianhydride (PMDA), *N*‐methyl‐2‐pyrrolidone (NMP), polyvinylidene fluoride (PVDF), and isoquinoline were purchased from Sigma Aldrich. Tetra‐(4‐aminophenyl)methane (TAPM) was bought from Manchester Organics, and mesitylene was obtained from Acros Organics. *N*,*N*‐Dimethylformamide and tetrahydrofuran were obtained from Fisher. Reduced graphene oxide (rGO) was obtained from Ossila (U.K.; Figure S4 shows the Raman spectrum of the rGO). All reagents were used as received without further purification.

**Physicochemical analyses**: FTIR spectra of the as‐prepared samples were measured on a Bruker alpha spectrometer by attenuated total reflectance. PXRD patterns were measured on Panalytical Empyrean diffractometer, equipped with a high‐throughput screening XYZ stage operating transmission mode, X‐ray focusing mirror, and PIXcel detector, using CuK_α_ (*λ*=1.541 Å) radiation. The morphology of the samples was studied on a Hitachi S‐4800 cold FE‐SEM. Samples were prepared by depositing the dry powders on 15 mm Hitachi M4 aluminum stubs using an adhesive high‐purity carbon tab before coating with a 2 nm layer of gold using an Emitech K550X automated sputter coater. Imaging was conducted at a working voltage of 10 kV and a working distance of 4 mm using a combination of upper and lower secondary electron detectors. Surface areas were measured by N_2_ adsorption and desorption at 77.3 K using a Micromeritics ASAP 2420 volumetric adsorption analyzer. The surface areas were calculated in the relative pressure range (*P*/*P* ^0^) from 0.05 to 0.12 using the *SA*_BET_ method. Samples were degassed at 100 °C for 15 h under vacuum (10–5 bar) before analysis. Ball milling was processed on Retsch MM400 with the Grinding jar sizes of 10 ml. Ex situ FTIR spectra were measured on a Nicolet iS50 FTIR spectrometer by attenuated total reflectance inside a glovebox under N_2_ atmosphere.

**Synthesis of PI**: A 10 mL Pyrex tube was charged with PMDA (43.6 mg, 0.2 mmol) and TAPM (38.1 mg, 0.1 mmol) in solution of NMP (0.2 mL), 1,3,5‐mesitylene (1.0 mL), and isoquinoline (0.02 mL). This reaction mixture was homogenized by ultrasonication for 10 min and the tube was flash frozen at 77 K (liquid N_2_ bath), degassed by three freeze‐pump‐thaw cycles, and evacuated to an internal pressure of 100 mTorr. The tube was sealed off and heated at 160 °C for 5 days to afford a yellow precipitate, which was isolated by filtration over a medium glass frit and washed with *N*,*N*‐dimethylformamide (50.0 mL) and tetrahydrofuran (50.0 mL). The product was dried under reduced pressure at 85 °C to give PI as a yellow powder (67 mg, 90 %). Anal. calcd. for (C_45_H_20_N_4_O_8_)_*n*_: C 72.5, H 2.68, N 7.52 %; found: C 65.85, H 3.53, N 8.03 %.

**Synthesis of PI*X***: A 10 mL Pyrex tube was charged with PMDA (43.6 mg, 0.2 mmol), TAPM (38.1 mg, 0.1 mmol) and rGO (10, 30, 50 wt % of the composite based on the yield of the PI) in solution of NMP (0.2 mL), 1,3,5‐mesitylene (1.0 mL), and isoquinoline (0.02 mL). This reaction mixture was homogenized by ultrasonication for 10 min and the tube was flash frozen at 77 K (liquid N_2_ bath), degassed by three freeze‐pump‐thaw cycles, and evacuated to an internal pressure of 100 mTorr. The tube was sealed off and heated at 160 °C for 5 days to afford a yellow precipitate, which was isolated by filtration over a medium glass frit and washed with *N*,*N*‐dimethylformamide (50.0 mL) and tetrahydrofuran (50.0 mL). The product was dried under reduced pressure at 85 °C to give PI*X* as a black powder. The yields calculated for PI10, PI30, and PI50 were 89.4, 88.7, and 87.5 %, respectively. Anal. calcd. for PI10: C 70.48, H 2.64, N, 8.18 %; found: C 64.45, H 3.01, N 7.00 %; Anal. calcd. for PI30: C 77.04, H 2.05, N 6.36 %; found: C 67.42, H 2.43, N 6.74 %; Anal. calcd. for PI50: C 83.60, H 1.47, N 4.50 %; found: C 68.91, H 1.96, N 6.70 %).

**Preparation of PI/rGO composite via mechanical ball milling**: PI (50 mg) and rGO (50 mg) were placed in a ball mill grinding jar together with seven steel balls (*Φ*=3 mm). The mixture was then ball milled at 30 Hz for 30 min. The product was recovered for the jar to give the physical mixture PI/rGO.

**Preparation of electrodes and electrochemical characterization**: The positive electrodes were prepared using the following method: PI/PI*X* active material, carbon black (Super C65, IMERYS) and PVDF were mixed in a 6 : 3 : 1 mass ratio. NMP was then added to generate a slurry. After stirring for 12 h a finely dispersed slurry was obtained, and this was coated onto an aluminum foil substrate (thickness×width×height=0.005×16×25 cm) using a doctor blade at a fixed thickness. The substrate was then dried at room temperature for 6 h followed by drying under vacuum pressure at 80 °C for 12 h giving the dry film. A punching tool was then used to punch out discs of 10 mm diameter, which were dried overnight under vacuum at 80 °C and transferred directly into a glovebox under an Ar atmosphere with H_2_O and O_2_ contents below 0.1 ppm. The resulting electrode loadings were in the range of 0.2–0.4 mg cm^−2^. Coin cells (CR2025) were then assembled inside the Ar‐filled glovebox. Lithium foil with a diameter of 12 mm and a thickness of 0.38 mm was used as the counter electrode, and glass microfiber (Whatman GF/F) was used as the separator. A mixture of DOL and DME (1 : 1 *v*/*v*) containing 1 m LiTFSI was used as the electrolyte (Duoduo Chemicals). After preparation, the coin cells were taken out of the glovebox and tested. CVs (Biologic) were recorded over a potential range of 1.5–3.4 V (vs. Li/Li^+^) at a scanning rate of 0.1 mV s^−1^, starting from open circuit potential (2.7 V) to the cathodic scan (discharge) direction. EIS (Biologic) was carried out over a frequency range from 1 MHz to 100 mHz, with an amplified voltage of 10 mV. Galvanostatic discharge‐charge cycling of the cells was measured within a potential window of 1.5–3.4 V (Maccor). All potentials are given versus Li/Li^+^. All electrochemical measurements were performed on the coin cells at 25 °C.

## Conflict of interest

The authors declare no conflict of interest.

## Supporting information

As a service to our authors and readers, this journal provides supporting information supplied by the authors. Such materials are peer reviewed and may be re‐organized for online delivery, but are not copy‐edited or typeset. Technical support issues arising from supporting information (other than missing files) should be addressed to the authors.

Supplementary
