# Supplementary material for: Crosslinked Polyimide and Reduced Graphene Oxide Composites as Long Cycle Life Positive Electrode for Lithium‐Ion Cells
Source: ChemSusChem. 2020 Sep 2;13(20):5571–9. doi: 10.1002/cssc.202001389 (PMC7693101; doi:10.1002/cssc.202001389)
Supplement: Supplementary file 1 — Supplementary [file CSSC-13-5571-s001.pdf]

ChemSusChem

Supporting Information

# Crosslinked Polyimide and Reduced Graphene Oxide Composites as Long Cycle Life Positive Electrode for Lithium-Ion Cells

Hui Gao, Bingbing Tian, Haofan Yang, Alex R. Neale, Marc A. Little, Reiner Sebastian Sprick, Laurence J. Hardwick,* and Andrew I. Cooper*© 2020 The Authors. Published by Wiley-VCH GmbH. This is an open access article under the terms of the Creative Commons Attribution License, which permits use, distribution and reproduction in any medium, provided the original work is properly cited.

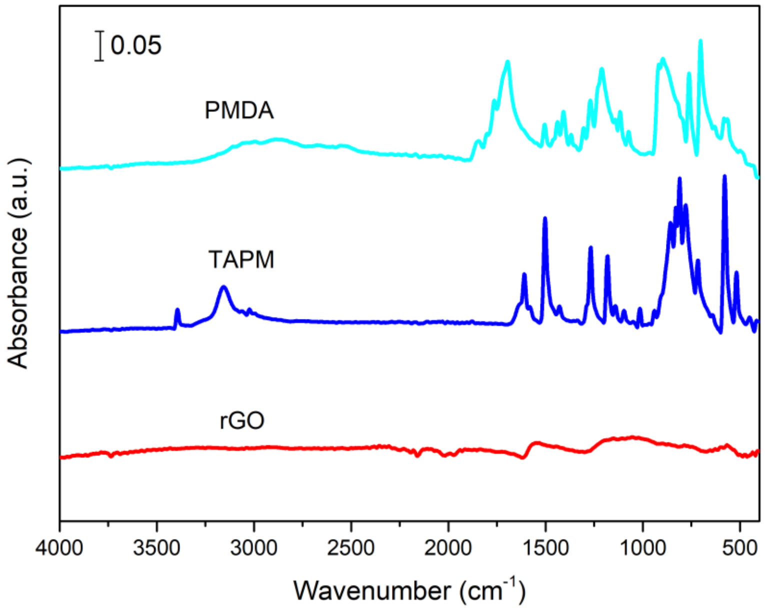


**Figure S1.** FT-IR Spectra of PMDA, TAPM, and rGO.

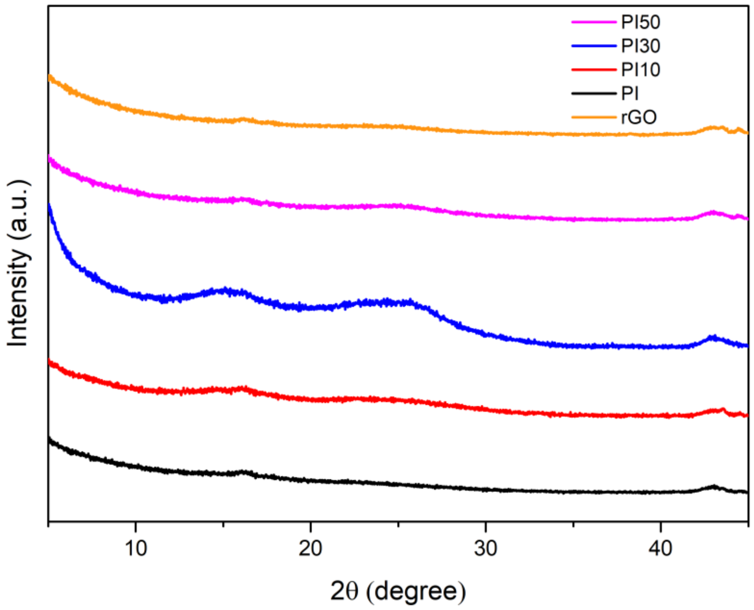


**Figure S2.** Amorphous PXRD patterns of **PI**, the **PIX** composites, and rGO.

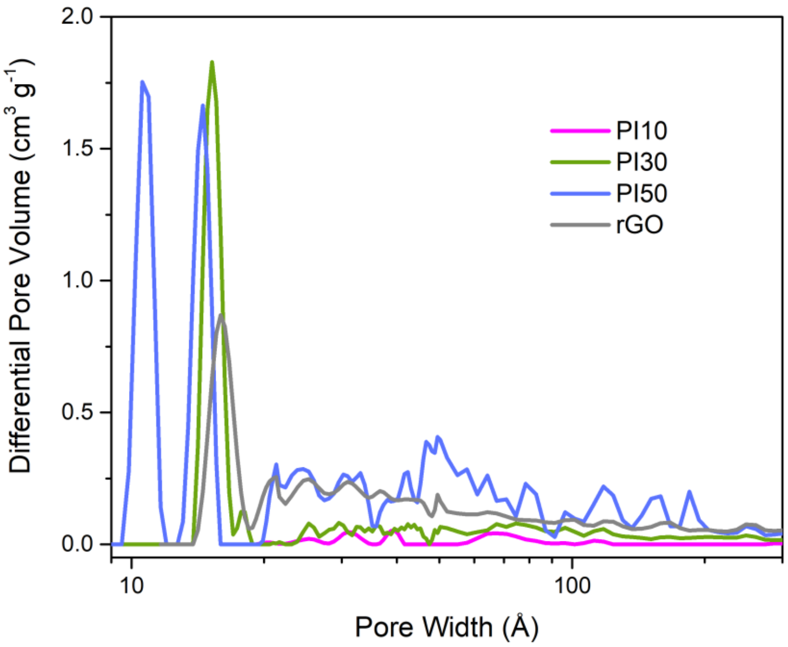


**Figure S3.** Pore size distribution plots of the **PIX** composites and rGO (calculated by NL-DFT for pillared clay).

**Table S1.** Surface areas and pore properties of **PI** and the **PIX** composites.

| **Sample** | ***SA*$_{BET}$[a] (m$^2$ g$^{-1}$)** | ***t*-plot micropore area[b] (m$^2$ g$^{-1}$)** | **Total pore volume[c] (cm$^3$ g$^{-1}$)** |
|---|---|---|---|
| PI | 6 | - | - |
| PI10 | 52 | 8 | 0.032 |
| PI30 | 233 | 95 | 0.143 |
| PI50 | 563 | 19 | 0.36 |

[a]Surface area calculated from the $N_2$ adsorption isotherms at 77.3 K using the BET equation; [b]Micropore surface area calculated from the nitrogen adsorption isotherms at 77.3 K using the *t*-plot equation; [c]Pore volume calculated from the nitrogen isotherm at $P/P_0$ = 0.99, 77.3 K.

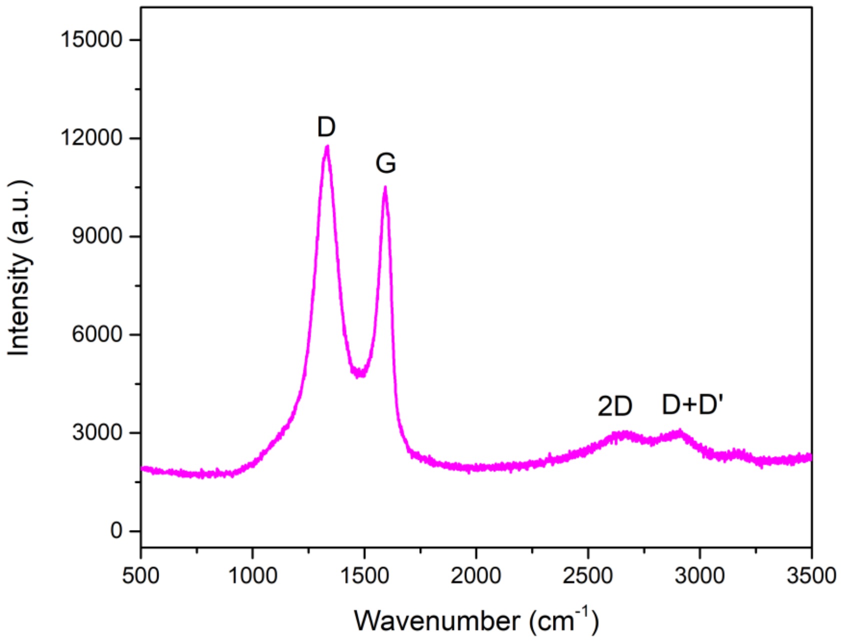


**Figure S4.** Raman spectrum of reduced graphene oxide.

**Theoretical specific capacity calculation**

Theoretical capacity C (mAh $g^{-1}$) was calculated using the following equation

$$C = \frac{N_A e}{3600\times(M_W/1000)} \qquad \text{Equation S1}$$

where $N_A e$ is the Faraday constant (96484 C $mol^{-1}$), and $M_w$ is the equivalent molecular weight of active materials that is defined as the molecular weight of the repeating unit cell of the polyimide or control compound divided with the number of electrons involved. The theoretical capacity is 144 mAh $g^{-1}$ calculated using Equation S1.

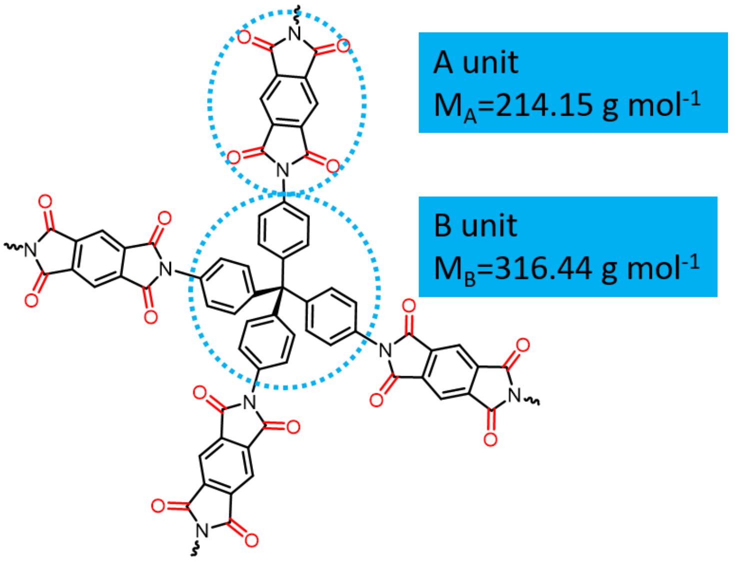


**Figure S5.** Chemical structure of **PI**.

The specific capacity of the **PIX** is calculated based on the mass of the polyimide in the composite. Both polyimide and rGO will, however, contribute to the capacity. So the total specific capacity of the **PIX** can be expressed as the following Equation

$$C_{\mathbf{PIX}} = \frac{a\times m\times C_{\mathrm{PI}}+\mathrm{b}\times m\times C_{\mathrm{rGO}}}{a\times m} \qquad \text{Equation S2}$$

Where, $C_{\mathbf{PIX}}$ and $C_{\mathbf{PI}}$ are the specific capacities of **PIX** and **PI,** respectively, in the composite electrodes. $C_{\mathrm{rGO}}$ is the specific capacity of pure rGO (48 mAh $g^{-1}$) without polyimide and *m* is the mass of the **PIX.** *a* and *b* are the contents of polyimide and rGO in the **PIX,** such that $a+b=1$. Therefore, the capacity contribution of rGO in the **PIX** is $C_{\mathbf{PIX}}$-$C_{\mathbf{PI}}$ = $(b\times C_{\mathrm{rGO}})/a$

Table S2. Capacity contribution of rGO in **PIX**.

| Sample | Calculation of capacity contribution of rGO in **PIX** / mAh $g^{-1}$ |
|---|---|
| **PI10** | 0.1×48/0.9 = 5 |
| **PI30** | 0.3×48/0.7 = 21 |
| **PI50** | 0.5×48/0.5 = 48 |

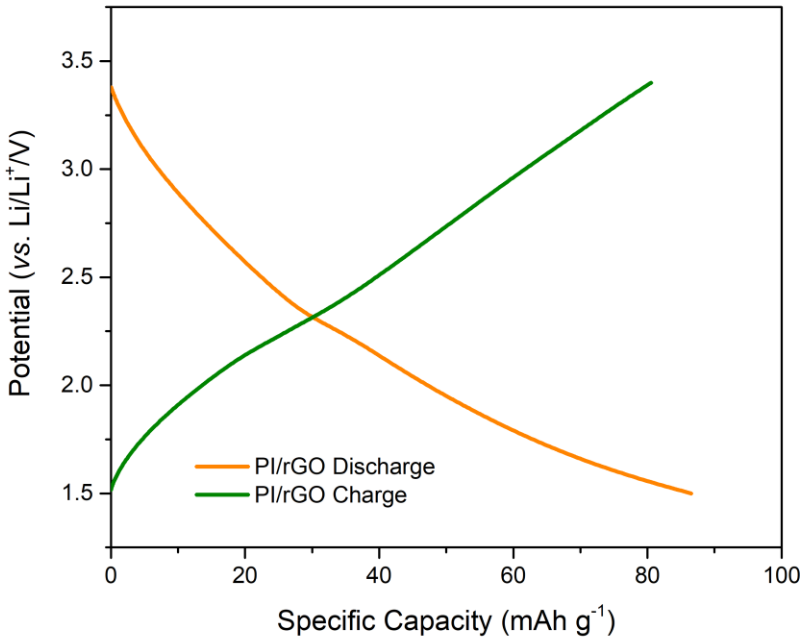


**Figure S6.** Galvanostatic charge/discharge curve of **PI**/rGO at 500 mA $g^{-1}$.

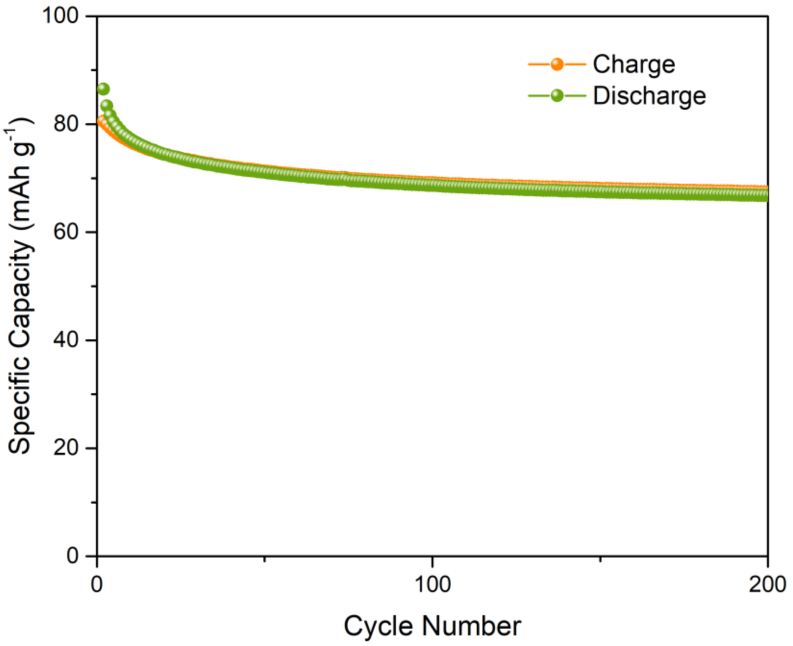


**Figure S7.** Cycling performance over 200 cycles for at **PI**/rGO at 500 mA $g^{-1}$.

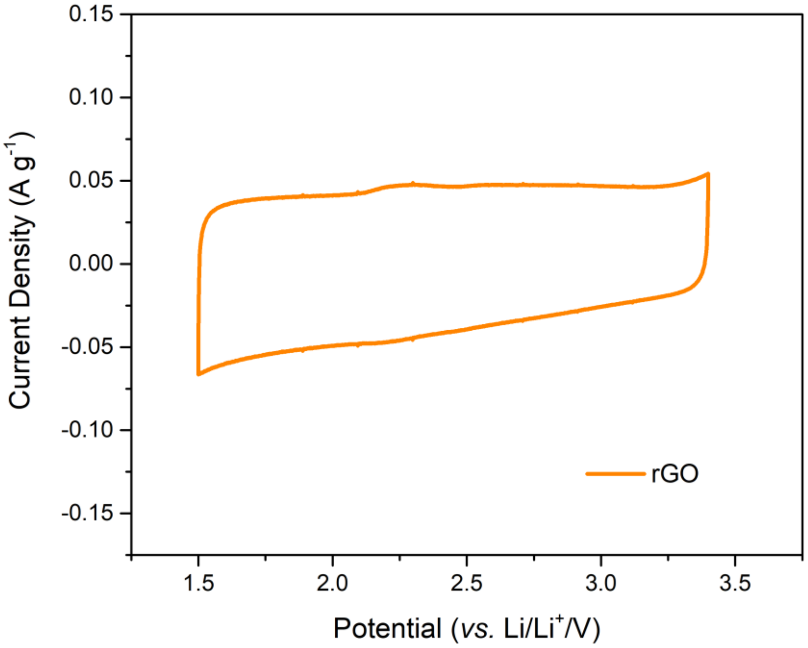


**Figure S8.** Cyclic voltammetry profile of rGO at a scan rate of 0.5 mV $s^{-1}$.

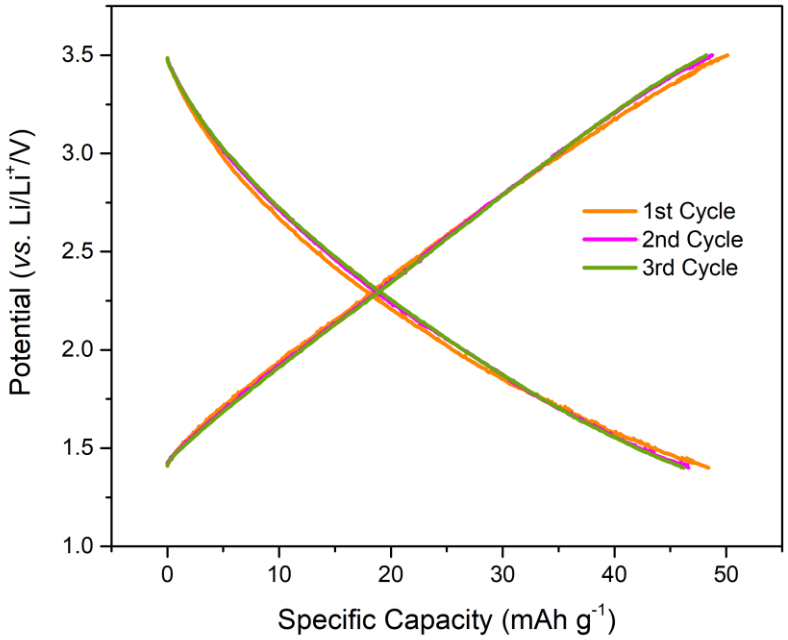


**Figure S9.** Galvanostatic charge/discharge curve of rGO at 500 mA $g^{-1}$.

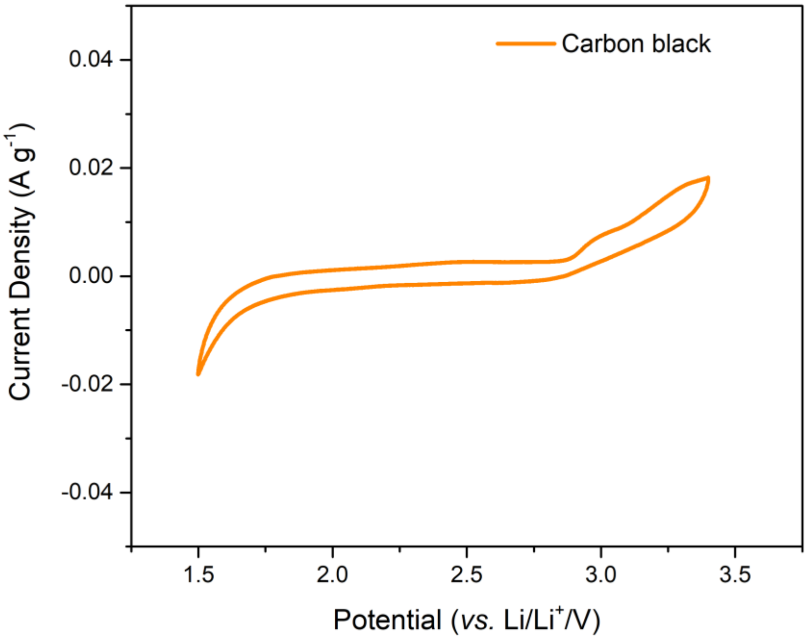


**Figure S10.** Cyclic voltammetry profile of Super C65 at a scan rate of 0.5 mV s$^{-1}$.

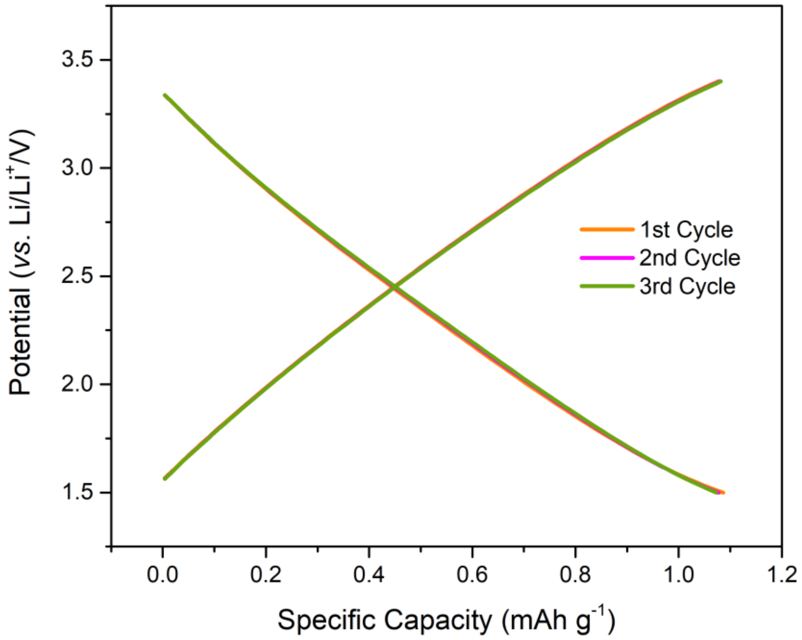


**Figure S11.** Galvanostatic charge/discharge curve of Super C65 at 500 mA g$^{-1}$.

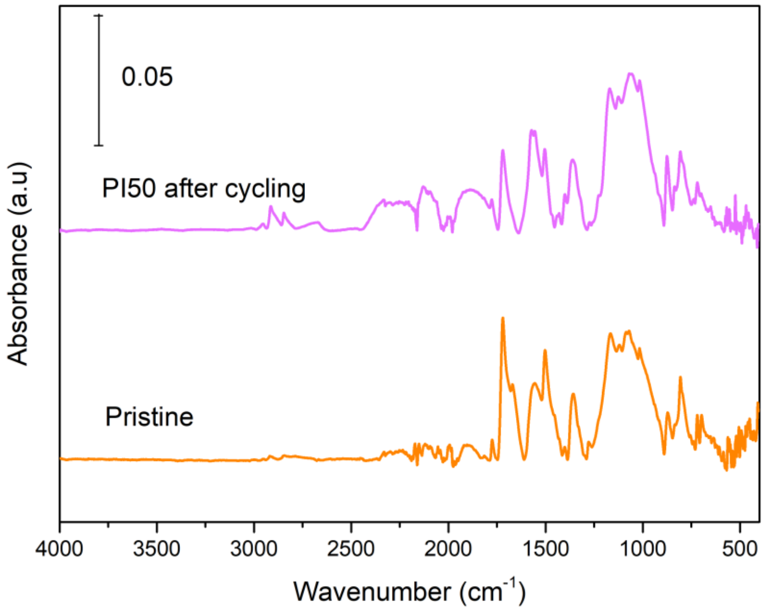


**Figure S12.** FT-IR Spectra of **PI50** electrode before and after 500 cycles.

**Table S3.** Battery performance comparison of **PIX50** with other reported PI cathodes.

| **Sample** | **Capacity Max (mAh $g^{-1}$)** | **Capacity Retention (%)** | **Symbol and Colour used in Figure 3b** | **Reference** |
|---|---|---|---|---|
| 2D-PAI@CNT | 104 at 0.96 C | 100 after 8000 cycles (20 C) | Purple sphere | Adv. Mater., 2019, 1901478 |
| PIDN-G | 270 at 0.1 C | 88.2 after 300 cycles (5 C) | Pink triangle | Angew. Chem. Int. Ed. Engl., 2018, 57 (30), 9443-9446. |
| PI-ECOF-1/rGO50 | 167 at 0.1 C | 76.1 after 300 cycles (1 C) | Orange Pentagon | Nanoscale, 2019, 11(12), 5330-5335. |
| PAQS-FGS-b | 165 at 0.1 C | 100 after 20 cycles (10 C) | Green square | Nano Lett., 2012, 12, 2205−2211 |
| PPy/r-GO | 74 at 1 C | 100 after 200 cycles (10 C) | Orange hexagon | Adv. Energy Mater. 2012, 2, 266–272 |
| G-g-PTMA/RGO | 466 at 200 mA $g^{-1}$ | 62 after 250 cycles (400 mA $g^{-1}$) | Cayno triangle | ACS Appl. Mater. Interfaces, 2016, 8, 17352−17359 |
| PTMA/graphene | 222 at 1 C | 57.1 after 20000 cycles (100 C) | Magnet triangle | Energy Environ. Sci., 2012, 5, 5221–5225 |
| VG 8/G-0.5 | 330 at 50 mA $g^{-1}$ | 81.9 after 100 cycles (100 mA $g^{-1}$) | Green diamond | Adv. Funct. Mater., 2017, 27, 1603603 |
| PI10G | 232.6 at 0.1 C | 80 after 1000 cycles (50 C) | Khaki diamond | ChemSusChem,2018,11,763–772 |
| HATNTA/GO | 410 at 50 mA $g^{-1}$ | 80 after 2000 cycles (500 mA $g^{-1}$) | Blue Pentagon | J. Mater. Chem. A, 2018, 6, 2752–2757 |
| PDI/RGO | 81.5 at 25 mA $g^{-1}$ | 73 after 500 cycles (25 mA $g^{-1}$) | Yellow circle | Electrochimica Acta, 2018, 282, 24-29 |
| NO-g-rGO | 310 at 0.5 C | 82.5 after 200 cycles (0.5 C) | Brown triangle | J. Mater. Chem. A, 2019, 7, 4438–4445 |
| **PIX50** | 172 at 500 mA $g^{-1}$ | 80 after 9000 cycles (2000 mA $g^{-1}$ =13.8 C) | Red star | This work |

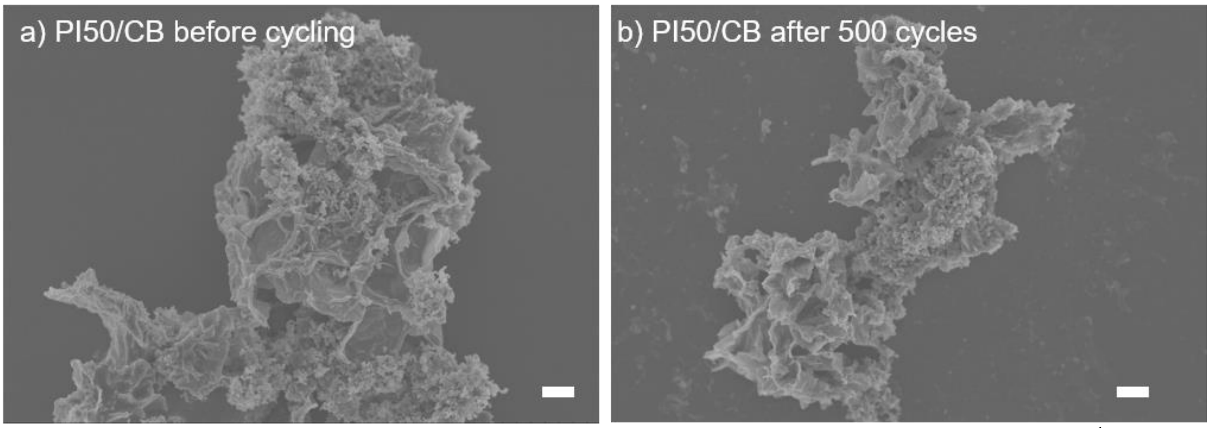


**Figure S13.** SEM Images of **PI50** electrode before and after 500 cycles at 500 mA $g^{-1}$ (scale bar: 1µm)

**Table S4.** The Coulombic efficiency of **PI** and **PIX** at different rate.

| Cycle Number | Current Density (mA $g^{-1}$) | **Coulombic Efficiency (%)** | | | |
|---|---|---|---|---|---|
| | | **PI** | **PI10** | **PI30** | **PI50** |
| 1 | 500 | 80.8 | 27.6 | 82.6 | 99.8 |
| 2 | | 107.1 | 93.8 | 105.7 | 99.4 |
| 3 | | 104.1 | 94.8 | 104.1 | 98.8 |
| 4 | | 102.8 | 95.7 | 103.0 | 100.4 |
| 5 | | 102.0 | 96.2 | 102.5 | 100.3 |
| 6 | 800 | 106.1 | 101.8 | 103.5 | 97.0 |
| 7 | | 101.1 | 98.1 | 101.3 | 95.2 |
| 8 | | 100.7 | 98.0 | 101.1 | 95.2 |
| 9 | | 100.5 | 98.0 | 101.0 | 95.1 |
| 10 | | 100.3 | 98.1 | 100.9 | 95.1 |
| 11 | 1000 | 102.4 | 100.2 | 101.7 | 96.9 |
| 12 | | 100.4 | 98.6 | 100.7 | 96.0 |
| 13 | | 100.1 | 98.5 | 100.6 | 96.0 |
| 14 | | 100.2 | 98.6 | 100.6 | 96.0 |
| 15 | | 100.1 | 98.6 | 100.5 | 96.0 |
| 16 | 1500 | 103.5 | 102.2 | 102.4 | 98.8 |
| 17 | | 100.4 | 99.3 | 100.6 | 97.1 |
| 18 | | 100.2 | 99.1 | 100.5 | 97.1 |
| 19 | | 100.0 | 99.1 | 100.5 | 97.0 |
| 20 | | 100.1 | 99.1 | 100.5 | 97.0 |
| 21 | 2000 | 102.1 | 101.4 | 101.7 | 99.0 |
| 22 | | 100.0 | 99.3 | 100.4 | 97.8 |
| 23 | | 100.2 | 99.3 | 100.3 | 97.7 |
| 24 | | 100.0 | 99.3 | 100.3 | 97.7 |
| 25 | | 100.0 | 99.2 | 100.3 | 97.7 |
| 26 | 500 | 89.5 | 89.4 | 95.8 | 99.6 |
| 27 | | 99.6 | 97.9 | 100.7 | 99.3 |
| 28 | | 99.9 | 98.1 | 100.7 | 99.0 |
| 29 | | 99.9 | 98.2 | 100.7 | 100.2 |
| 30 | | 100.0 | 98.2 | 100.6 | 99.6 |

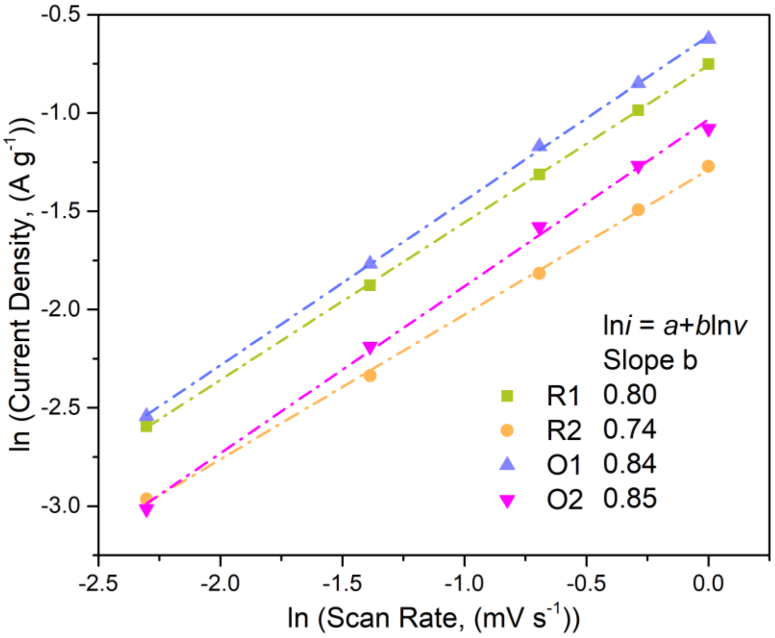


**Figure S14.** ln *i* vs. ln *v* plots to determine the b values of different peaks based on the subtracted data.

a)

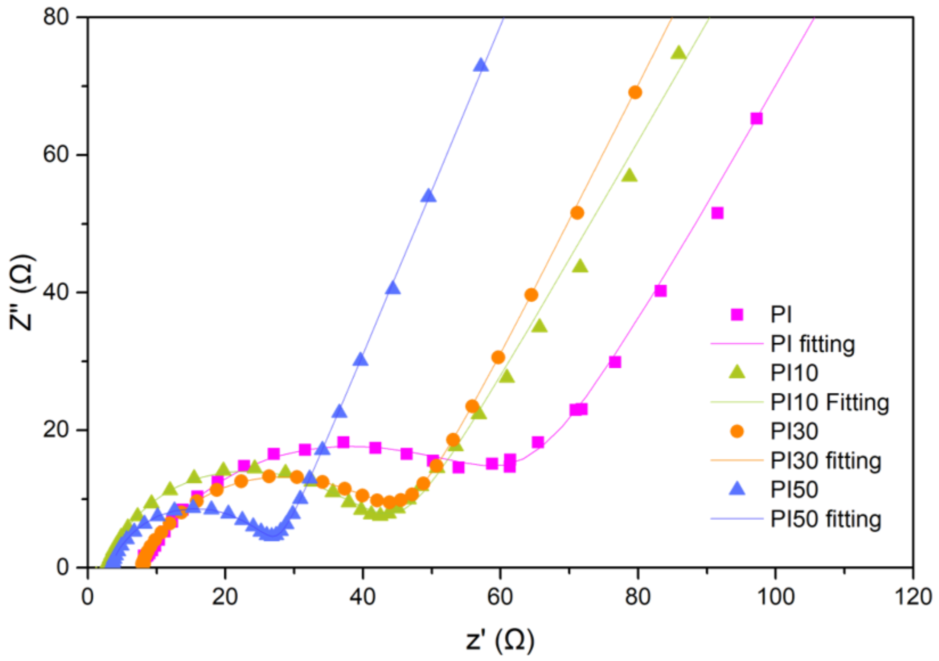


b)

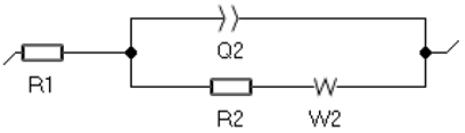


**Figure S15.** a) EIS spectra of **PI** and the **PIX** composite electrodes; b) Equivalent circuit used for fitting the electrochemical impedance data. The $R_s$ (R1), $R_c$ (R2), CPE (Q2) and W (W2) represent cell solution resistance, charge-transfer resistance, constant phase element and Warburg impedance.

**Table S5.** Fitted values of elements in the equivalent circuit for EIS data of **PI** and the **PIX** composite electrodes.

| | **$R_s$ (Ω)** | **CPE (F)** | **$R_c$ (Ω)** | **W(Ω)** |
|---|---|---|---|---|
| **PI** | 8.1 | $22.9\times10^{-6}$ | 47.7 | 26.3 |
| **PI10** | 2.7 | $24.1\times10^{-6}$ | 38.1 | 4.3 |
| **PI30** | 7.7 | $37.8\times10^{-6}$ | 35.4 | 10.3 |
| **PI50** | 3.3 | $17.7\times10^{-6}$ | 22.1 | 4.2 |
